# Cross-sectional analysis of financial relationships between board certified allergists and the pharmaceutical industry in Japan

**DOI:** 10.1186/s12910-024-01014-2

**Published:** 2024-02-20

**Authors:** Anju Murayama, Yuki Senoo

**Affiliations:** 1 School of Medicine, Tohoku University, 2-1 Seiryo-machi, Aoba ward, Sendai City, Miyagi 980-0872 Japan; 2 Department of Population Health Science and Policy, Icahn School of Medicine at Mount Sinai, New York City, NY USA; 3 Higashi Totsuka Memorial Hospital, Yokohama City, Kanagawa Japan

**Keywords:** Conflicts of interest, Industry payments, Ethics, Japan, Japanese Society of Allergology

## Abstract

**Background:**

Financial interactions between pharmaceutical companies and physicians lead to conflicts of interest. This study examines the extent and trends of non-research payments made by pharmaceutical companies to board-certified allergists in Japan between 2016 and 2020.

**Methods:**

A retrospective analysis of disclosed payment data from pharmaceutical companies affiliated with the Japanese Pharmaceutical Manufacturers Association was conducted. The study focused on non-research payments for lecturing, consulting, and manuscript drafting made to board-certified allergists from 2016 to 2020. We performed descriptive analyses on payment data. Trends were analyzed using generalized estimating equation models.

**Results:**

Of the 3,943 board-certified allergists, 2,398 (60.8%) received non-research payments totaling $43.4 million over five years. Lecturing fees comprised 85.7% ($37.2 million) of the total payment amounts. For allergists who received at least one payment, the median amount per allergist was $3,106 (interquartile range: $966 – $12,124), in contrast to a mean of $18,092 (standard deviation: $49,233) over the five-year span. The top 1% and 10% of these allergists accounted for 20.8% and 68.8% of all non-research payments, respectively. The annual payment amounts significantly increased by 7.2% annual increase (95% CI: 4.4 – 10.0%, *p* < 0.001) each year until 2019, but saw a significant decrease in 2020 amid the COVID-19 pandemic.

**Conclusion:**

The majority of allergists received non-research payments, with a notable concentration among a small group. Payments increased annually until the pandemic’s onset, which coincided with a substantial decrease. Further research is needed to explore the implications of these financial interactions on clinical practice and patient care in Japan.

## Introduction

Pharmaceutical companies frequently provide payments to physicians for both research and non-research activities [1, 2]. Such financial interactions, while often instrumental in fostering healthcare innovation and improving patient care, also lead to conflicts of interest [3], potentially biasing physicians’ clinical practice [4–8]. In Japan, pharmaceutical companies have been reported to make substantial non-research payments totaling $1.8 billion, with $1.1 billion allocated for the sponsorship of conferences and lectures geared toward drug promotion, and $236.0 million distributed for lecture and consulting services [9]. The majority of these lecture and consulting payments were directly made to individual physicians. Previous studies suggest that the incidence of non-research payments among Japanese physicians [10–17] surpasses that in other developed countries such as the United States, Australia, and France [1, 18–24].

The discipline of allergy and clinical immunology has been marked by the introduction of numerous novel biologics for the treatment of allergic diseases, including asthma, atopic dermatitis, and allergic rhinitis. Concurrently, the last decade in Japan has witnessed the approval of new therapeutic agents for allergic rhinitis, such as antihistamines (e.g., rupatadine, desloratadine, and bilastine) and sublingual immunotherapy products. The introduction of these novel drugs has expanded the therapeutic choices available to physicians and patients, yet the absence of comparative clinical trials assessing these drugs has resulted in increased promotional activities by pharmaceutical companies. Prior research indicates a yearly 7.2% increase in marketing payments to allergists in the United States, rising from $13.1 million in 2014 to $19.2 million in 2019 [25]. Additionally, these payments were associated with the prescribing patterns of new biologics for moderate and severe asthma among allergists in the United States [26]. The evaluation of the financial relationships between allergists and pharmaceutical companies is therefore of particular importance. However, data on these relationships in Japan remain sparse. This study aims to assess the extent of financial interactions between allergists and pharmaceutical companies in Japan using publicly disclosed payment data.

## Methods

### Study design setting, and participants

We conducted a retrospective analysis of payment data publicly disclosed by major pharmaceutical companies in Japan. The aim was to examine the extent and trends of personal payments made to all board-certified allergists in the country. The analysis included all allergists certified by the Japanese Society of Allergology as of February 2022. The Society, established in 1952, is the preeminent professional organization for medical researchers and physicians specializing in allergy in Japan and is the sole credentialing body for allergists in the nation. As of February 2022, a total of 3943 allergists were recognized as board-certified by the Society [27].

### Payment data disclosed by pharmaceutical companies

To improve transparency in financial relationships between pharmaceutical companies and healthcare professionals, the Japanese Pharmaceutical Manufacturers Association (JPMA), representing over 80 leading pharmaceutical companies, mandates its members to disclose payments for lecturing, consulting, and manuscript drafting made to physicians, including the recipients’ names and affiliations on their websites, as previously explained [9, 28]. These payment data, disclosed by all JPMA member firms, are collected by an independent research organization and structured into a searchable public online payment database (Yen For Docs, https://yenfordocs.jp/en) since 2016. However, according to the JPMA guidance, payment categories, such as meals, travel and accommodations, and other gifts, are disclosed in aggregated amounts and could not be analyzed at individual physician level. Additionally, payments for lecturing, consulting, and drafting are generally paid directly to physicians from pharmaceutical companies and in larger amounts than payments for other categories [18, 29].

### Data collection and coding

We retrieved the names and affiliated hospitals of all 3943 board-certified allergists from the Japanese Society of Allergology’s website. We extracted all non-research payments for lecturing, consulting, and drafting services to these allergists by JPMA-affiliated companies from 2016 to 2020 from the public payment database [30], in line with methodologies used in prior studies [31, 32]. Instances of allergists with identical names were resolved by manual searches via Google and verification through official hospital and organizational websites, as previously described [33]. We excluded payments to individuals who could not be verified as board-certified allergists or were confirmed as ineligible physicians through our search process from our study samples.

### Statistical analyses

We calculated mean and median payments per allergist and proportion of allergists receiving payments. We assessed the concentration of payments among allergists using the Gini index, a measure traditionally applied to analyze income inequality in economics [34, 35]. The index ranges from 0 (indicating uniform payment distribution) to 1 (where a single allergist receives all payments), with higher values signaling greater disparity. We also analyzed payment data by category and the pharmaceutical companies making these payments. Trends in the number of allergists receiving payments and the payment amounts from 2016 to 2020 were evaluated using generalized estimating equation (GEE) models. Due to the non-normal distribution of payments, the analyses were conducted using a log-linked GEE model with a Poisson distribution and a negative binomial GEE model [11–13, 32]. The study period was bifurcated into two intervals (2016–2019 and 2020) to determine the impact of the COVID-19 pandemic on payments, as indicated by a notable reduction in payments to physicians in the United States in 2020 [20, 25, 36]. A sensitivity analysis was also conducted for companies that consistently made payments over the five-year span. For trend analysis, we adjusted for inflation, converting all payment values to their 2020-Japanese yen equivalent. Statistical significance was set at a p-value of less than 0.05.

### Ethical clearance

As all data used in this study were publicly available and met the definition of non-human subjects research, institutional review board approval and informed consent from the study participants were not required.

## Results

### Summary of non-research payments to board-certified allergists

**Fig. 1:**
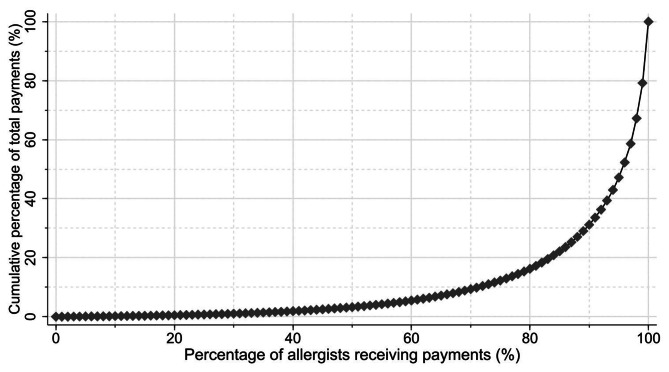
Concentration of non-research payments to allergists between 2016 and 2020

Between 2016 and 2020, 2,398 of the 3,943 board-certified allergists (60.8%) received at least one non-research payment for lecturing, consulting, and drafting from pharmaceutical companies, as detailed in Table 1. The total number of payments amounted to 30,849, with an aggregate value of $43,385,284, distributed by 84 different pharmaceutical entities over the five years. Lecturing payments constituted the majority of this sum, exceeding $37.2 million (85.7% of the total payment amounts), followed by consulting at $4.3 million (9.9%) and drafting at $1.9 million (4.3%). There was large gap between mean and median payment amounts. For allergists who received at least one payment, the median amount per allergist was $3,106 (interquartile range [IQR]: $966 – $12,124), in contrast to a mean of $18,092 (standard deviation [SD]: $49,233) over the five-year span. The Gini index, used to measure payment distribution among allergists, was 0.874, suggesting that a small proportion of allergists received the majority of non-research payments over the five years. Specifically, the top 1%, 5%, and 10% of these allergists accounted for 20.8%, 52.8%, and 68.8% of all non-research payments, respectively (Fig. 1).

**Table 1:** Summary of non-research payments to board-certified allergists

Variables	Value
Total amounts of payments	
Payment values, $	43,385,284
Number of payments, No.	30,849
Number of companies making payments, No.	84
Payments per allergist	
Mean (standard deviation) ^a^	
Payment values, $	18,092 (49,233)
Number of payments, No.	12.9 (17.7)
Median (interquartile range) ^a^	
Payment values, $	3,106 (966–12,124)
Number of payments, No.	6.0 (2.0–16.0)
Maximum^a^	
Payment values, $	611,190
Number of payments, No.	161.0
Gini index	0.874
Allergists with specific amount of payments (*N* = 3943), n (%)	
No payment	1545 (39.2)
$1-$1,000	615 (15.6)
$1,001-$5,000	802 (20.3)
$5,001-$10,000	297 (7.5)
$10,001-$50,000	485 (12.3)
$50,001-$100,000	100 (2.5)
$100,001-$200,000	57 (1.5)
$200,001-	42 (1.1)

Legends: ^a^ Payments per allergist were calculated among allergists who received one or more payments, as 39.2% of allergists did not receive any payments over the five years

### Payments by company

Table 2 presents the distribution of payments and amounts by the top 10 companies. Of the 84 companies contributing payments, the foremost 10 and 20 companies, by total payment magnitude, were responsible for 64.6% ($28.0 million) and 83.8% ($36.4 million) of all payments within the 2016 to 2020 timeframe. AstraZeneca made the largest total payments amounting to 14.0% of all payments ($6.1 million) in monetary value, followed by Boehringer Ingelheim Japan ($4.1 million, 9.4%), Novartis Pharma ($3.2 million, 7.4%), Kyorin Pharmaceutical ($2.6 million, 6.0%), and Mitsubishi Tanabe Pharma ($2.4 million, 5.6%). Regarding the number of allergists receiving payments, Kyorin Pharmaceutical was at the forefront, providing payments to 994 allergists (25.2% of all allergists), with AstraZeneca following at 884 allergists (22.4%), and Novartis Pharma at 799 allergists (20.3%), over the span of five years.

**Table 2:** Payments by top 10 companies making the largest payments to allergists between 2016 and 2020

Ranking	Company name	Amounts of payments (%), $	Number of payments (%), No.	Number of allergists receiving payments (%), n
1st	AstraZeneca	6,056,278 (14.0)	2839 (9.2)	884 (22.4)
2nd	Boehringer Ingelheim Japan	4,071,844 (9.4)	2139 (6.9)	717 (18.2)
3rd	Novartis Pharma	3,205,803 (7.4)	1919 (6.2)	799 (20.3)
4th	Kyorin Pharmaceutical	2,585,524 (6.0)	2241 (7.3)	994 (25.2)
5th	Mitsubishi Tanabe Pharma	2,434,910 (5.6)	1354(4.4)	589 (14.9)
6th	Taiho Pharmaceutical	2,044,290 (4.7)	1491 (4.8)	623 (15.8)
7th	Sanofi	2,038,459 (4.7)	1292 (4.2)	599 (15.2)
8th	GlaxoSmithKline	2,026,210 (4.7)	1012 (3.3)	516 (13.1)
9th	Torii Pharmaceutical	1,841,077 (4.2)	1222 (4.0)	500 (12.7)
10th	Maruho	1,706,618 (3.9)	1037 (3.4)	409 (10.4)

Legend: The ranking was based on the monetary amounts of non-research payments between 2016 and 2020

### Trends in non-research payments to allergists between 2016 and 2020

The aggregate sum of non-research payments to allergists showed an increased trend from $7.8 million in 2016 to $10.0 million in 2019 but there was a decrease to $8.4 million in 2020 (Table 3). During the study period from 2016 to 2019, 40.3–42.2% of all allergists received non-research payments from pharmaceutical companies, whereas this proportion contracted to 36.0% (1418 allergists) in 2020. The median annual payments to those allergists who received payments increased from $1,332 in 2016 to $1,526 in 2019. The payments per allergist each year significantly increased by 7.7% (95% CI: 5.0–10.6, *p* < 0.001) from 2016 to 2019. There was no significant trend in the number of allergists receiving payments during the same period. Contrarily, there was a significant reduction of 14.8% (95% CI: -17.9% to -11.5%, *p* < 0.001) in the number of allergists receiving payments and a 21.2% decrease (95% CI: -25.4% to -16.8%, *p* < 0.001) in payments per allergist in 2020 when compared to those between 2016 and 2019.

**Table 3:** Trend in personal payments from pharmaceutical companies to board-certified allergists between 2016 and 2020

Variables	2016	2017	2018	2019	2020	Average relative yearly change between 2016 and 2019 (95% CI), %	Relative change rate between 2016–2019 and 2020 (95% CI), %
All pharmaceutical companies							
Total payments, $	7,821,861	8,274,237	8,953,824	9,981,069	8,354,293	–	–
Payments per allergist							
Mean (standard deviation), $	4,819 (11,395)	5,204 (12,305)	5,517 (12,200)	6,002 (13,243)	5,892 (13,200)	7.7 (5.0–10.6)*	-21.2 (-25.4 to -16.8)*
Median (interquartile range), $	1,332 (521–3,898)	1,435 (521–3,974)	1,526 (521–4,521)	1,535 (539–5,021)	1,518 (539–4,674)		
Maximum, $	167,946	155,035	125,560	154,826	131,500		
Physicians with payments (%), n	1623 (41.2)	1590 (40.3)	1623 (41.2)	1663 (42.2)	1418 (36.0)	0.9 (-0.1 to 2.1)	-14.8 (-17.9 to -11.5)*
Gini index	0.888	0.890	0.888	0.885	0.904	–	–
Pharmaceutical companies making payments throughout five years							
Total payments, $	7,645,832	8,192,889	8,710,193	9,653,679	8,144,163	–	–
Payments per allergist							
Mean (standard deviation), $	4,782 (11,289)	5,208 (12,254)	5,468 (12,085)	6,015 (13,280)	5,919 (13,171)	7.2 (4.4–10.0)*	-20.4 (-24.6 to -15.9)*
Median (interquartile range), $	1,317 (521–3,873)	1448 (521–3,990)	1,526 (521–4,495)	1,564 (547 − 4,931)	1,526 (539–4,666)		
Maximum, $	166,980	155,035	125,560	154,826	130,757		
Physicians with payments (%), n	1599 (40.6)	1573 (39.9)	1593 (40.4)	1605 (40.7)	1376 (34.9)	0.2 (-0.9 to 1.3)	-14.8 (-17.4 to -10.7)*
Gini index	0.889	0.891	0.889	0.889	0.906	–	–

Abbreviations: standard deviation (SD); interquartile range (IQR),95% confidence interval (95% CI). * *p* < 0.001

In sensitivity analyses that only included data from the 54 companies making consistent payments over the five-year period, there was a 7.2% annual increase (95% CI: 4.4 – 10.0%, *p* < 0.001) in payments per allergist from 2016 to 2019. Yet, in 2020, both the number of allergists receiving payments and the payment amounts per allergist decreased significantly by 14.8% (95% CI: -17.4% to -10.7%, *p* < 0.001) and 20.4% (95% CI: -24.6% to -15.9%, *p* < 0.001), respectively.

## Discussion

### Summary of principal findings

This comprehensive longitudinal cross-sectional study scrutinized the financial relationships between pharmaceutical companies and all allergists certified by the Japanese Society of Allergology for non-research activities from 2016 to 2020. To the best of our knowledge, this research is the first study to assess the size and trends of non-research reimbursements to physicians from pharmaceutical companies for services such as lecturing, consulting, and manuscript drafting in the field of allergology and clinical immunology in Japan. The analysis revealed that 60.8% of all allergists received non-research payments totaling $43.4 million (over 4.6 billion yen) across the five-year span. Notably, these payments escalated by more than 7% each year in the pre-pandemic period, with the largest payments from companies that manufacture and market medications for allergic conditions including allergic rhinitis, asthma, and atopic dermatitis in Japan.

### Comparison with previous studies

This investigation found that approximately 40% of allergists received annual non-research payments from the pharmaceutical industry, with over 60% compensated across a five-year period for activities such as lecturing, consulting, and drafting. These figures are consistent with prior research conducted in Japan. The lowest annual proportions of physicians receiving such payments were observed among otolaryngologists (24.3–26.0%) and the highest among rheumatologists (49.8–52.5%), as documented across 10 clinical specialties [11–15, 17, 31–33, 37]. Concurrently, the annual median payments received by allergists, ranging from $1,332 to $1,526, were within the range reported for other internal medicine subspecialties. Gastroenterologists received $829 to $946 [13], pulmonologists $1085 to $1428 [15], hematologists $1241 to $1629 [33], infectious disease specialists $1430 to $1737 [14], and rheumatologists $1544 to $1635 annually [11]. Thus, the financial interactions between the pharmaceutical industry and board-certified allergists do not appear to be markedly distinct compared to those in other medical fields.

The analysis revealed annual non-research payments to allergists in the range of $7.8 million to $10.0 million. This contrasts with previous research in the United States, which recorded annual payments between $10.6 million and $18.8 million for similar activities among allergists and clinical immunologists [25, 26]. Considering the healthcare expenditure of approximately $4.3 trillion in the United States compared to Japan’s $403.4 billion, the non-research payments to Japanese allergists appear relatively substantial. This disparity may be attributable to the high prevalence of allergic diseases in Japan, estimated at 49.2% for allergic rhinitis [38], compared to 15-30% in the United States [39–41].

During the period from 2014 to 2018, several novel drugs for allergic rhinitis were approved in Japan. Kyorin Pharmaceutical, ranked fourth among the top payers, released desloratadine (Desalex) in 2016. Furthermore, four of the top ten payers introduced new second-generation antihistamines, including rupatadine fumarate (Rupafin by Mitsubishi Tanabe Pharma, approved in 2017), bilastine (Bilanoa by Taiho Pharmaceutical, approved in 2016), and fexofenadine hydrochloride/pseudoephedrine hydrochloride (Dellegra by Sanofi, approved in 2012). The first sublingual immunotherapy product for cedar pollen allergy, Cedartolen by Torii Pharmaceutical, was approved in 2014 for patients aged 12 years and older. Additionally, Miticure, another product by Torii Pharmaceutical for dust mite allergy, received initial approval in 2015 for patients over 12 years and was later extended to younger patients in 2018 [42]. The introduction of these novel drugs and the expansion of treatment options for allergic rhinitis are likely contributors to the high volume of payments and the increasing trend observed from 2016 to 2019.

The investigation also revealed that a disproportionate amount of non-research payments was concentrated among a small number of allergists, consistent with findings across various specialties [1, 11–14, 21, 32]. Pharmaceutical companies frequently engage physicians recognized for their clinical expertise and research contributions to deliver lectures to their peers. These key opinion leaders often receive substantial non-research payments as compensation for activities such as lecturing, consultations, and drafting manuscripts and pamphlets [43–45]. While collaboration between these physicians and pharmaceutical companies is instrumental in advancing drug and medical product development, such significant financial ties may pose conflicts of interest, particularly for physicians in positions demanding high ethical standards, such as board members of professional medical societies [10, 13, 23, 46], editors of medical journals [47–49], authors of clinical guidelines [16, 35, 50, 51], and members of governmental advisory boards [52–56]. Conflicts of interest in these influential roles may introduce bias in decision-making, potentially compromising the quality of patient care [57, 58]. Future research should evaluate the characteristics of allergists receiving the most significant payments and examine the impact of these financial associations on their clinical and policy decisions.

The study identified a notable reduction in non-research payments to allergists in Japan in 2020, coinciding with the onset of the COVID-19 pandemic. Payments to allergists declined by over 20% in terms of amounts per allergist during this year. Comparative studies in the United States documented a significant drop in non-research payments across various specialties [19, 20, 36, 59–63], while research payments remained unaffected during the pandemic [18, 29, 62–66]. Notably, the reduction rate in the personal payments to allergists were lower in our study than those reported among allergists in the United States. Previous research reported that personal payments to allergists for speaking compensation decreased by 53.1% in 2020 than those in 2019 in the United States [25]. The COVID-19 pandemic led to the cancellation or deferral of numerous academic conferences and restricted activities of pharmaceutical representatives within healthcare facilities in Japan. As the background reasons for the lower reduction in the payments during the pandemic in Japan are unclear, further research is needed to understand the nature of financial relationships between physicians and the pharmaceutical industry in Japan.

This investigation is the first to demonstrate the pandemic’s impact on financial interactions between physicians and the pharmaceutical industry in Japan, supported by a substantial data disclosed by the pharmaceutical industry. However, the implications of this decline in financial payments on the influence of the industry over physicians’ clinical decisions remain unclear. Previous studies have suggested associations between payments to allergists and their prescribing behaviors [7, 8, 66, 67], particularly concerning new asthma biologics in the United States [66]. Future research is essential to explore the relationship between physician payments and clinical practice and to assess how the pandemic has affected the pharmaceutical industry’s influence on clinical practice in Japan.

This study has several limitations. Firstly, the analysis was confined to payment data from JPMA-affiliated pharmaceutical companies, potentially overlooking financial relationships between allergists and non-member companies. However, it is important to note that JPMA-affiliated companies represent over 80% of the market share for drugs and medical products in Japan [68]. Additionally, non-member companies do not publicly disclose payment information to allergists. Consequently, the impact of undisclosed financial relationships with non-member companies is likely minimal. Secondly, the payment data are self-reported by the companies in compliance with JPMA guidelines, which do not impose penalties for non-adherence, raising concerns about the accuracy of the disclosed data. Thirdly, the Japanese Society of Allergology publishes only the most recent list of board-certified allergists; therefore, the study may include some allergists who were not board-certified during the payment period. Finally, the JPMA transparency guidance does not require their member companies to disclose detailed information of payments such as associated drug names, drug category, and dates of payments. Therefore, our study could not analyze the specific drugs associated with the personal payments to allergists.

## Conclusion

In conclusion, this investigation revealed that over 60% of allergists certified by the Japanese Society of Allergology received non-research payments related to lecturing, consulting, and manuscript drafting from pharmaceutical companies between 2016 and 2020. These payments were predominantly distributed to a limited number of allergists. Notably, there was a significant annual increase in payment amounts exceeding 7% prior to the pandemic. In contrast, a marked decline in both the number of allergists receiving payments and the total payment amounts was observed in 2020, concurrent with the onset of the COVID-19 pandemic in Japan.

## Data Availability

All payment data used in this study were extracted from a publicly available database, Yen For Docs database (https://yenfordocs.jp/). Due to the privacy restriction, the datasets generated during and/or analyzed during the current study are available from the corresponding author on reasonable request.

## Abbreviations

95% CI: 95% confidence interval
GEE: Generalized estimating equation
IQR: Interquartile range
JPMA: Japanese Pharmaceutical Manufacturers Association
